# Enhancing Immunoglobulin G Goat Colostrum Determination Using Color-Based Techniques and Data Science

**DOI:** 10.3390/ani15010031

**Published:** 2024-12-26

**Authors:** Manuel Betancor-Sánchez, Marta González-Cabrera, Antonio Morales-delaNuez, Lorenzo E. Hernández-Castellano, Anastasio Argüello, Noemí Castro

**Affiliations:** IUSA-ONEHEALTH 4, Animal Production and Biotechnology, Institute of Animal Health and Food Safety, Universidad de Las Palmas de Gran Canaria, Campus Montaña Cardones, 35413 Arucas, Spain; manuel.betancor105@alu.ulpgc.es (M.B.-S.); marta.gonzalezcabrera@ulpgc.es (M.G.-C.); antonio.moralesdelanuez@ulpgc.es (A.M.-d.); lorenzo.hernandez@ulpgc.es (L.E.H.-C.); noemi.castro@ulpgc.es (N.C.)

**Keywords:** colostrum, immunoglobulin G, machine learning, deep learning, decision tree, neural network, IgG prediction

## Abstract

Circulating immunoglobulin G (IgG) concentrations in newborn goat kids are not sufficient to protect the animal against external agents. Therefore, consumption of colostrum, rich in immune components, shortly after birth is crucial. Traditional laboratory methods used to measure IgG concentrations, such as ELISA or RID, are reliable but costly and impractical for many farmers. This study proposes a more accessible alternative for farmers to predict IgG concentration in goat colostrum by integrating color-based techniques with machine learning models, specifically decision trees and neural networks, through the development of two regression models based on colostrum color data from Majorera dairy goats. A total of 813 colostrum samples were collected in a previous study (June 1997–April 2003) that utilized multiple regression analysis as a reference to verify that applying data science techniques improves accuracy and reliability. The decision tree model outperformed the neural network, achieving higher accuracy and lower error rates. Both models provided predictions that closely matched IgG concentrations obtained by ELISA. Therefore, this methodology offers a practical and affordable solution for the on-farm assessment of colostrum quality (i.e., IgG concentration). This approach could significantly improve farm management practices, ensuring better health outcomes in newborn animals by facilitating timely and accurate colostrum quality evaluation.

## Simple Summary

Newborn goat kids rely on colostrum intake to gain immunity, as the circulating antibodies at birth are not enough to face infectious diseases. Colostrum contains vital proteins like immunoglobulin G (IgG), which protect newborn animals from diseases, as the immune system is not able to synthesize enough antibodies yet. Traditional methods used to determine IgG concentrations are often expensive and not accessible to many farmers. This study explores an affordable solution to predict IgG concentration in goat colostrum by using a color-based method combined with artificial intelligence. By measuring colostrum color and using machine learning models, such as decision trees and neural networks, an accurate prediction method was developed as a practical method to be used on farms. These novel models provide similar results to those obtained using expensive laboratory tests but at a much lower cost. This method could help farmers make better decisions to enhance newborn goat health status, and consequently improve animal welfare, reducing production costs and increasing economic profits.

## 1. Introduction

Newborn goat kids are born with an immature immune system that seems to be unable to produce its own antibodies [1], mainly immunoglobulin G (IgG), to protect them against external agents. In addition, in this species, there is a limited transplacental transfer of maternal antibodies [2]. In ruminants, the transfer of IgG and other antibodies is performed through colostrum intake during the period immediately postpartum [1]. This process, known as transfer of passive immunity (TPI), is vital for providing immediate protection to newborn goat kids until their immune system starts producing endogenous antibodies [3,4].

Colostrum is a secretion synthesized by the mammary gland during the last eight weeks of gestation [1]. Besides its nutritional function, colostrum contains high concentration of immunoglobulins, and other bioactive compounds such as oligosaccharides, lactoferrin, lysozyme, and some other minor proteins [5] that enhance the immunological development and gastrointestinal health of the offspring. Previous research shows that colostrum composition changes rapidly, within the first 10 to 24 hours following parturition. Thus, the prompt determination of colostrum quality to ensure proper TPI to the newborn goat kid becomes essential [6].

For the quantification of IgG, different laboratory techniques can be used, such as ELISA (enzyme-linked immunosorbent assay) or RID (radial immunodiffusion) [7]. These methods provide a concentration value for IgG that is valid for controlling the quality of colostrum, but the associated cost and complexity are not suitable for most goat farmers.

On the other hand, the most used techniques on the farm are the colostrometer and the Brix refractometer [8,9,10,11]. Although the Brix refractometer and colostrometer can provide an approximate indication of colostrum quality, they lack precision. Direct measurement of IgG concentration remains the most reliable method for assessing the passive immunity provided by colostrum. This is particularly crucial due to external factors, such as the difficulty of maintaining a consistent 20°C when using the colostrometer, which may not always be feasible during colostrum evaluation on farms [12].

Argüello et al. [13] described a novel farm method for assessing IgG concentration in goat colostrum, based on color analysis. They identified a correlation between colostrum color and IgG concentration using conventional statistical techniques. This new method is an important step forward that adds to current farming techniques, though it has not yet reached lab-level precision.

Since 2005, artificial intelligence (AI) has advanced exponentially. Algorithmic advancements, the exponential increases in computing power and storage, and an explosion of data, as highlighted in the McKinsey AI guide [14], have evolved synergistically. This evolution has facilitated access to computational and statistical tools, offering new possibilities for the scientific community.

The utilization of machine learning and deep learning techniques in the field of healthcare and animal production has yielded highly promising results, as evidenced by several authors in recent years [15,16,17]. These techniques have added value to the datasets held by scientific laboratories, enriching knowledge with a fresh perspective from an alternative point of view.

The objective of this study is to integrate AI techniques to predict IgG concentration in goat colostrum by evaluating a color-based method for testing IgG concentration. It will employ both machine learning and deep learning methodologies, employing regression based on decision trees and neural networks, respectively. The goal is to find models that can provide more reliable support in goat farms, using a method that can be implemented at a significantly lower cost than traditional laboratory techniques, without sacrificing a high level of accuracy and reliability.

## 2. Materials and Methods

### 2.1. Dataset

The data used in the analyses were collected between June 1997 and April 2003, as detailed in the article published in 2005 [13]. A total of 813 colostrum samples were analyzed. Color and IgG concentration were measured from the first and second milking of the dams, which were performed using a milking machine. The samples were collected from Majorera goats located on four dairy farms in the Canary Islands, Spain.

Serum IgG concentration was determined using the single radial immunodiffusion method [7].

Color data were acquired using a Minolta CR200 Chromameter (Aquatecnis, Madrid, Spain). The color data registered with the Chromameter was CIE L*a*b*, where L* represents relative lightness, a* indicates relative redness, and b* represents relative yellowness [18]. These data were then transformed into CIE L*C*h format for the dataset, which includes the Chroma indicating color purity or intensity, and the hue represented by an angle in a circular color space. CIE L*C*h is a cylindrical representation of the CIE L*a*b* color space, which facilitates a more intuitive understanding of color, in addition to allowing a more direct and meaningful comparison in perceptual terms [19].

For the transformation of values into Chroma, the following formula was used:

$$C^{*}=\sqrt{{\left(a^{*}\right)}^{2}+{\left(b^{*}\right)}^{2}}$$

Similarly, the arctangent $h=atan2(a^{*},b^{*})$ was used for the calculation of hue angle value [20]. 

The dataset was randomly shuffled, and subsequently, two new datasets were generated: one for training and one for testing. The training set comprised 80% of the data, with a total of 650 records, while the test set comprised 20%, with a total of 163 records. A random seed was used during the data split to ensure reproducibility of the results. The use of a seed guarantees that the same training and testing subsets are generated each time the experiment is run This allows for consistent comparisons across different models and runs, as well as facilitating debugging and replication in future studies [21].

### 2.2. Models

In this study, two regression models were selected, one from the field of machine learning and the other from deep learning, to compare their performance against traditional statistical methods. The goal was to assess whether these advanced techniques offered improvements in predictive accuracy and model robustness. By focusing on widely used approaches in each category, a balanced evaluation of their effectiveness can be provided.

For the machine learning approach, decision trees were chosen, as they represent one of the most established and interpretable models in this category [22]. On the deep learning side, a neural network was selected, as it is one of the most used architectures for handling complex data patterns in regression tasks [23]. These models were selected to ensure that the comparison reflects the most representative techniques in each field [24].

The selected models are regression-based, even though a subsequent factorization will be performed to allow for a comparison similar to that conducted in the original study.

#### 2.2.1. Decision Trees

Decision Trees are a widely used machine learning technique for decision-making and predictive modelling [22]. The term “Decision Tree” arises from the graphical representation of the model, which resembles an inverted tree. In this structure, each internal node represents a decision based on a specific attribute, each branch corresponds to the outcome of that decision, and each terminal node (leaf) represents a predicted outcome or class.

Decision trees can be divided into two main types: classification trees and regression trees. Classification trees are used to assign data points to discrete categories, whereas regression trees are employed to predict continuous values.

The construction of a decision tree begins with a dataset composed of input variables (features), which can be either numerical or categorical, and output variables (labels), representing the predicted outcome. The algorithm’s first step is to identify the optimal feature to split the data. This optimal feature is the one that results in the most homogeneous grouping of data points within the branches.

The specific splitting criterion depends on the type of decision tree. For classification tasks, common criteria include Gini impurity or entropy, while for regression tasks, the criterion typically used is mean squared error (MSE). Once the best feature has been selected, the dataset is divided into two or more branches, and this process is applied recursively to each branch.

The tree continues to split the data until a predefined stopping criterion is met. These criteria may include reaching a point where all data points in a branch belong to the same class (in classification tasks), the number of data points in a branch falling below a minimum threshold, or the tree reaching its maximum allowed depth. When further splitting is no longer possible, the branch becomes a leaf node, which provides the final output of the decision tree.

For this study, regression trees were applied, and the tree splitting was performed using mean squared error (MSE) as the criterion to minimize prediction errors.

Given that the dataset used in this study was small and well-balanced, no maximum tree depth was set, allowing for the algorithm to determine the optimal tree depth. However, a minimum depth of two branches was specified to ensure sufficient complexity. Additionally, data balancing techniques were not deemed necessary due to the balanced nature of the dataset.

Lastly, pre-pruning and post-pruning techniques [25] were not applied, as they were not necessary for the scale and characteristics of the data used in this study.

#### 2.2.2. Neural Network

Neural networks are a deep learning technique inspired by the architecture and functioning of the human brain [26]. These computational models are composed of basic units called artificial neurons, which are organized into layers. Each neuron receives inputs, processes the information through a predefined mathematical function (known as an activation function), and generates an output.

Neural networks are organized into three distinct types of layers. The input layer serves as the entry point for the raw data, passing it along to the subsequent layers for further processing. Following the input layer are the hidden layers, which are responsible for the bulk of the network’s processing. Each hidden layer takes the output of the previous layer as its input, applying a series of transformations to capture patterns in the data. Finally, the output layer produces the network’s final result, which can either be a predicted value (in the case of regression tasks) or a classification outcome (for classification tasks).

For this study, a feedforward neural network (FNN) was selected, as it was determined to be the most appropriate model given the structure and characteristics of the dataset [27]. The feedforward architecture ensures efficient forward propagation of data without the complexity of recurrent connections, making it well-suited for tabular and structured datasets like the one used in this study.

Additionally, in deep learning, a normalization function was generated using standard scaling from the training data, as there were no outlier values significantly affecting the mean or standard deviation of the sample [28]. This normalization function was subsequently applied to both the training and test datasets before being fed into the neural network model, ensuring that the data were properly scaled for effective training and performance.

The neural network was designed with several specific characteristics tailored to the dataset. Kernel initialization was performed using a normal distribution, as this method was deemed most appropriate given the dataset’s properties. The network architecture includes four hidden layers, structured as follows: the first layer contains 64 neurons, the second and third layers contain 128 neurons each, and the fourth layer consists of 64 neurons.

To enable the network to learn complex, non-linear relationships in the data, a rectified linear unit (ReLU) activation function was used for all hidden layers [29]. Additionally, L2 regularization was applied to layers two, three, and four, with a regularization factor of 0.01, to reduce the risk of overfitting [30]. The output layer consists of a single neuron, responsible for generating the regression value for the predictive task.

The network’s performance was optimized using MSE as the loss function, which is appropriate for regression tasks, as it calculates the average of the squared differences between predicted and actual values. RMSprop, a gradient-based optimization algorithm, was employed to adjust the model’s weights. This algorithm incorporates a moving average of squared gradients to prevent large oscillations during training, and the learning rate was set to 0.001 for gradual and efficient weight updates.

The model was trained for 2000 epochs, with each epoch representing a full pass over the training data. During each epoch, the network’s weights were updated to minimize the loss function. An early stopping mechanism was also implemented, halting training if no improvement in performance was observed over 200 consecutive epochs. This prevented overfitting and reduced unnecessary computation time.

**Figure 1:**
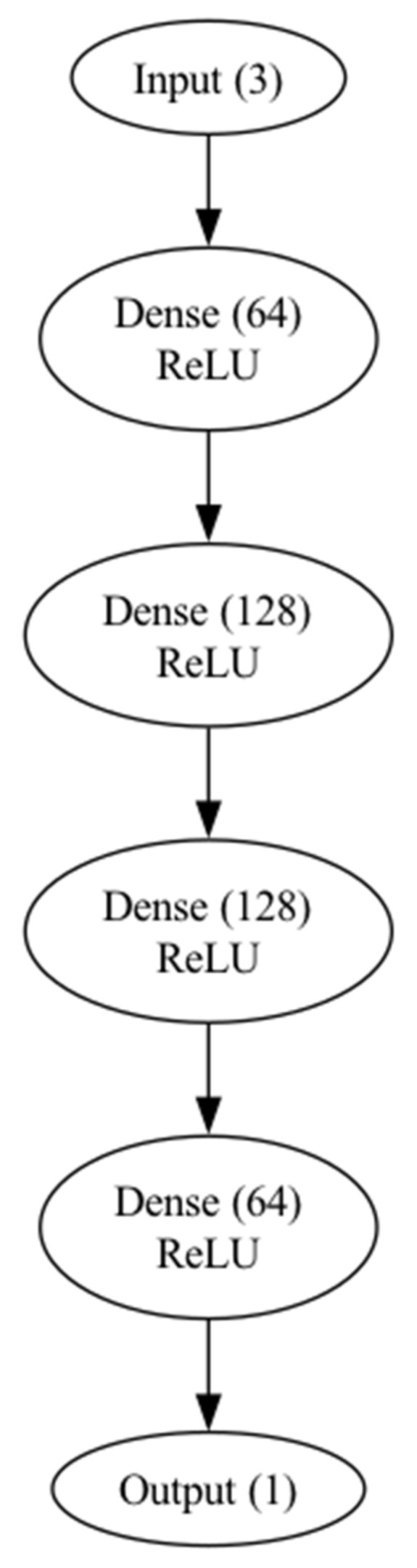
Architecture of the feedforward neural network.

Figure 1 illustrates a schematic representation of the different layers in the feedforward neural network used in this study. Each layer is labeled with the number of neurons it contains and the activation function applied.

### 2.3. Performance Evaluation

To assess model performance, different techniques were employed during the training phase and in the final analysis of the results. Specifically, MSE and root mean squared error (RMSE) were used during the initial phase, while mean absolute error (MAE) and the coefficient of determination (r^2^) were applied to the final predictions [31].

The combination of these two techniques provides a more comprehensive view of model performance. During training, MSE was used to penalize larger errors more heavily, which helped both models, decision trees and neural networks, focus on minimizing significant deviations, thus enhancing the optimization process. Once the predictive values were obtained, MAE allowed for a more straightforward interpretation of the results, as it is expressed in the same units as the predicted values. This combination is effective in providing a more balanced and detailed understanding of model quality.

For comparison with the previous study, a factorization of the target and predicted values was also performed, following the protocol outlined in the original research [13]. Specifically, IgG values exceeding 20 mg/mL were categorized as HIGH, while the remaining values were classified as LOW.

Once the factorization was performed, several comparative metrics were utilized to assess the performance of the current study in comparison to the original. These included the contingency tables, which provides a summary of prediction results, and accuracy, a measure of the overall correctness of the model. Sensitivity (or recall) and specificity were also analyzed to evaluate the model’s ability to correctly identify positive and negative cases, respectively. Additionally, the negative predictive value (NPV) was used to assess the likelihood that negative predictions were correct [32]. Finally, the ROC-AUC (area under the curve) was employed to evaluate the model’s discriminative ability across different thresholds [33].

### 2.4. Tools and Development Environment

Computational experiments were conducted using Python 3.9.6 [34] in a locally hosted environment. Python was chosen for its versatility and the availability of extensive libraries that facilitated the implementation of both machine learning and deep learning algorithms, as well as data processing tasks. Scikit-learn 1.4.2 was used for the machine learning model [35], and TensorFlow 2.16.2 [36] alongside Keras 3.4.1 [37] was employed for the deep learning model. This setup ensured efficient handling of the computational workload, providing a reliable and stable environment for model development and testing.

## 3. Results

The results of this study are presented in two sections: The first section focuses on the outcomes derived from two predictive methods: decision trees and neural networks. These models were applied to the objective data, and their performance was evaluated using quantitative metrics, including MAE, MSE, RMSE, and r^2^. These metrics provide a comprehensive assessment of how accurately each model predicts the given data.

In the second section, the results are factorized to generate data that can be compared to those presented in the original study. This factorization allows for a more refined analysis of the classification outcomes. Key metrics such as accuracy, precision, recall, NPV, and the ROC-AUC are presented to evaluate the performance of the models in a classification context.

### 3.1. Predictive Model Performance Evaluation

In Table A1, the final color values in the CIE L*C*h format (L, Cr, and Hue) for each of the test dataset records are provided, along with the original IgG values [13] and the predicted values from the regression models. Specifically, the predicted IgG values from the decision tree model are shown in the IgG_p_DT column, while the predicted values from the neural network model are presented in the IgG_p_NN column.

The performance of the regression models was initially evaluated using the MSE and RMSE. The decision tree model achieved an MSE of 3.6571, corresponding to an RMSE of 1.9124. In comparison, the neural network model produced a higher MSE of 5.1804, resulting in an RMSE of 2.2761.

Additionally, the models were assessed using the MAE. The decision tree model achieved a MAE of 0.3206, while the neural network model showed a considerably higher MAE of 1.1076.

Furthermore, the r^2^ coefficients were calculated to assess the proportion of variance explained by each model. The decision tree model achieved an r^2^ of 0.9644, indicating that it explains 96.44% of the variance in the data. The neural network model obtained a slightly lower r^2^ of 0.9541, explaining 95.41% of the variance.

**Figure 2:**
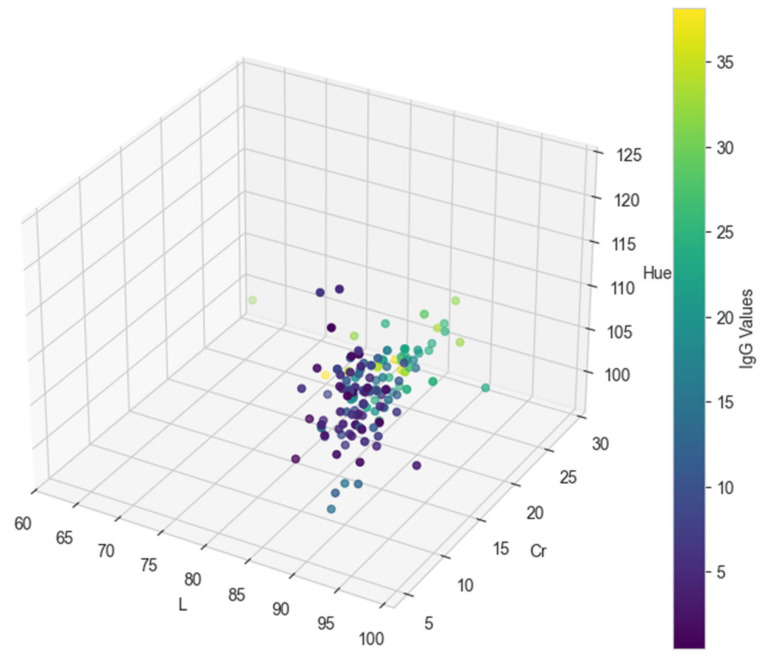
Three-dimensional plot of IgG values based on L, Cr, and Hue.

**Figure 3:**
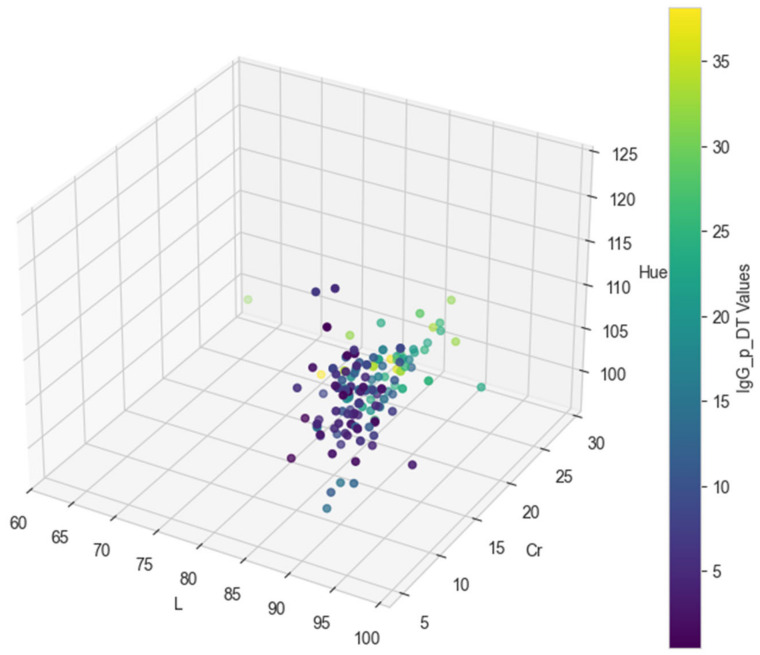
Three-dimensional plot of decision tree prediction values based on L, Cr, and Hue.

**Figure 4:**
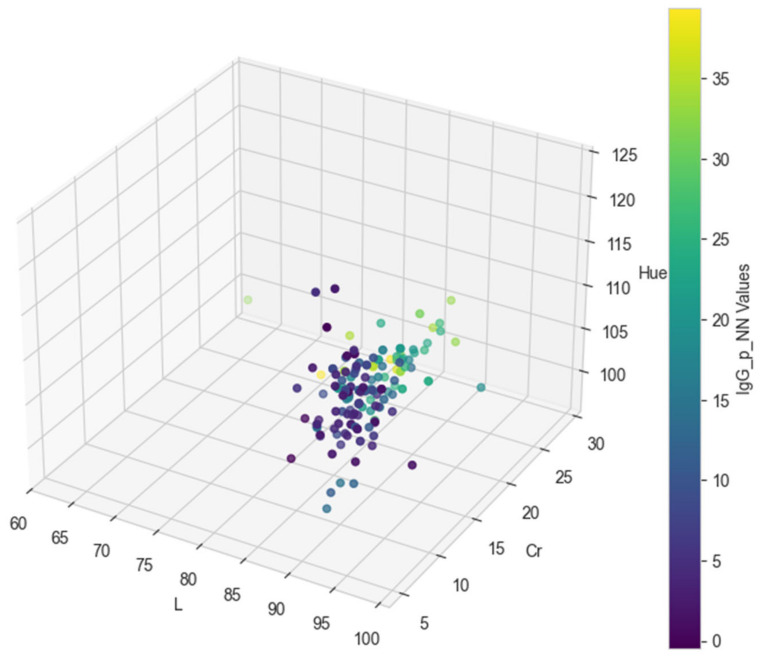
Three-dimensional plot of neural network prediction values based on L, Cr, and Hue.

Figure 2, Figure 3 and Figure 4 visually represent the original and predicted data values from the regression models. The scatter plots illustrate the relationship between the original IgG values and the predicted values generated by both the decision tree and neural network models, using the final color values in the CIE L*C*h format (L, Cr, and Hue) as coordinates. These figures provide a clear comparison of model performance and predictive accuracy.

### 3.2. Classification Metrics After Factorization

In this section, both the target and predicted values were factorized, classifying any value greater than 20 mg/mL of colostrum IgG as HIGH and the remaining values as LOW. This transformation allows for the application of a series of classification metrics, facilitating a direct comparison with the results presented in the original article. By categorizing the values into these two distinct classes. The aim is to assess the models’ performance in distinguishing between high and low IgG levels, providing a more detailed evaluation of their predictive capabilities in line with the classification approach used in previous studies.

Table 1 and Table 2 display the contingency tables generated from the factorization process for both the decision tree and neural network models, respectively. These matrixes provide a detailed overview of the models’ classification performance, illustrating the number of correct and incorrect predictions for the HIGH and LOW categories. The values obtained from these contingency tables will be used in the subsequent calculation of performance metrics, allowing for a more comprehensive comparison of the models’ classification accuracy based on the established threshold.

The decision tree model achieved an overall accuracy of 0.9816. The model demonstrated a sensitivity of 1.0 and a specificity of 0.9741. Additionally, NPV was 1.0, indicating that all predictions classified as LOW were accurate.

The neural network model achieved an overall accuracy of 0.9632. The model demonstrated a sensitivity of 0.94 and a specificity of 0.9735. Additionally, NPV was 0.9735.

**Figure 5:**
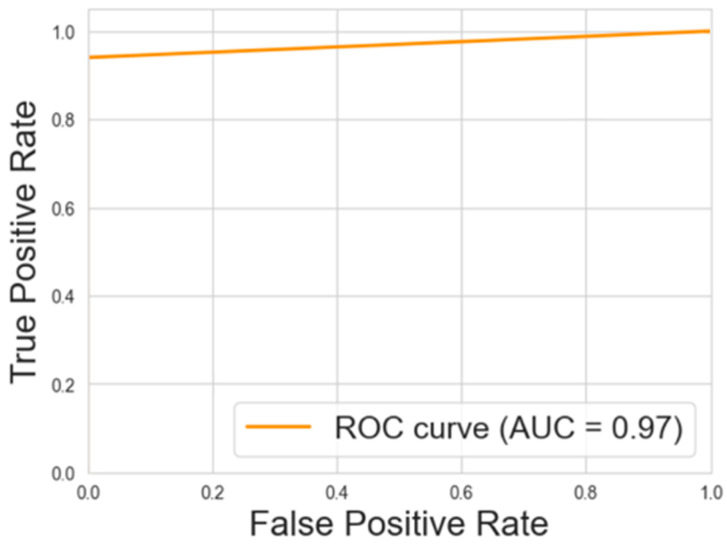
ROC-AUCs for the decision tree regression model.

**Figure 6:**
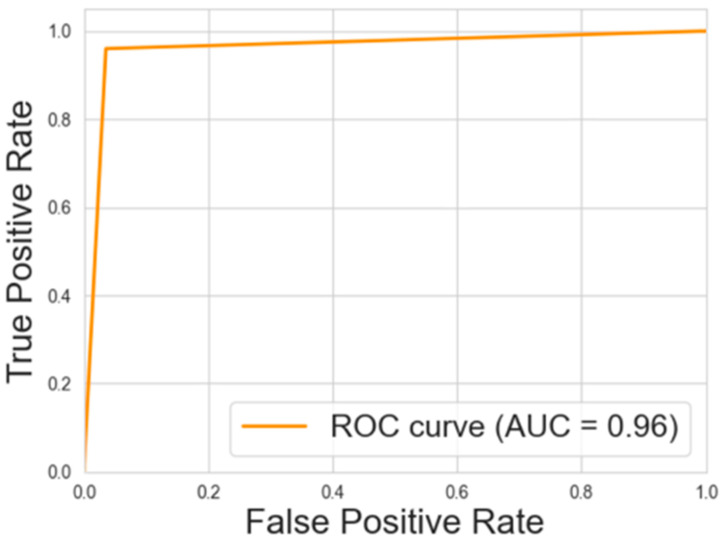
ROC-AUC curves for the neural network regression model.

Figure 5 and Figure 6 show the ROC-AUCs for the decision tree and neural network models, respectively. 

## 4. Discussion

### 4.1. Results Overview

The evaluation of the models’ performance, as detailed in Table A1, reveals notable differences between the decision tree and neural network models in terms of error metrics and explanatory power. Analyzing the MSE and RMSE, the decision tree model demonstrated better performance with an MSE of 3.6571 and an RMSE of 1.9124, compared to the neural network’s higher MSE of 5.1804 and RMSE of 2.2761. These values indicate that the decision tree model makes more accurate predictions overall, producing lower deviations from the actual IgG values.

The MAE further supports this observation, with the decision tree model showing lower MAE of 0.3206, while the neural network model yielded a higher MAE of 1.1076. This disparity suggests that the decision tree model consistently produces smaller prediction errors, while the neural network model exhibits greater variability in its predictions.

Importantly, considering the MAE in relation to the expected IgG concentration range in colostrum, the relatively low error rates of both models indicate that their precision approaches that of laboratory techniques. The decision tree’s low MAE suggests that its predictions are highly accurate, making it a practical tool for being used in farm settings where quick and reliable IgG estimates are needed. Although the neural network model has a higher MAE, its accuracy is still within a range that could be useful for practical applications in livestock management.

In terms of the coefficient of determination, which measures the proportion of variance explained by the models, both models performed well, although the decision tree model again outperformed the neural network. The decision tree model achieved an r^2^ of 0.9644, while the neural network model explained 95.41% (r^2^ = 0.9541). While this difference is small, it indicates that the decision tree model captures the underlying patterns in the data more effectively.

In addition to the evaluation of the continuous predictions, the target and predicted values were factorized into two categories (i.e., HIGH and LOW) setting as a threshold IgG concentration in colostrum (i.e., 20 mg/mL). This binary classification enabled the application of various classification metrics, aligning with the approach used in the original study and facilitating direct comparisons. The transformation provided clearer assessment of the models’ ability to distinguish between high and low IgG concentrations, which is particularly important for practical decision-making in farm environments.

The contingency tables generated for both the decision tree and neural network models offer a detailed view of their classification performance. These tables summarize the correct and incorrect classifications for each category (i.e., HIGH and LOW), allowing for a straightforward calculation of the models’ accuracy, sensitivity, specificity, and other key metrics. The decision tree model demonstrated near-perfect classification performance, with an accuracy of 98.16%, meaning that almost all predictions were correct. Additionally, its high sensitivity (1.0) underscores its ability to identify high IgG cases without error, while its specificity of 0.9741 indicates the ability to identify low IgG cases. The negative predictive value (NPV) of 1.0 further highlights the reliability of the decision tree model in predicting LOW cases, suggesting that no false negatives occurred in this category.

The neural network model also performed well, achieving an overall accuracy of 96.32%. While its sensitivity (0.94) was slightly lower than that of the decision tree, it still correctly identified 94% of the high IgG cases. Its specificity (0.9735) and NPV (0.9735) were comparable to the decision tree, indicating a similar performance in identifying low IgG cases and avoiding false negatives. Despite the marginal differences between the two models, both demonstrated strong predictive power, with very few misclassifications across both categories.

The ROC-AUCs, presented in Figure 5 and Figure 6, provide an additional layer of evaluation for these models. The decision tree model achieved an AUC of 0.97, while the neural network model followed closely with an AUC of 0.96. These high AUC values reflect the models’ excellent ability to distinguish between the HIGH and LOW categories across a range of thresholds, further reinforcing the robustness of the classification models.

Overall, the results from both regression and classification analyses suggest that the decision tree model offers better predictive accuracy and consistency compared to the neural network model. The decision tree’s lower error rates and higher r^2^ value indicate its superior ability to capture the nuances of the dataset, while the neural network’s higher error rates suggest a greater susceptibility to overfitting. Nevertheless, both models demonstrate strong predictive power, explaining over 95% of the variance and showing remarkable precision, particularly in terms of MAE, making them viable for practical implementation in farm environments, where their predictive accuracy closely mirrors that of laboratory methods.

Furthermore, the factorization of values and subsequent application of classification metrics reinforce the decision tree model’s edge in performance, particularly in terms of sensitivity and NPV, which are critical for accurately identifying high IgG cases. Despite this advantage, the neural network model also performed admirably, offering comparable specificity and overall strong classification capabilities. Both models present reliable and robust tools for distinguishing between high and low IgG levels, a crucial task for optimizing farm management and improving animal health outcomes.

The dataset used in this study exclusively comprises data from Majorera goats located on four dairy farms in the Canary Islands, Spain. This specificity was necessary to ensure a direct and reliable comparison with previous studies using similar methodologies. However, the methods presented in this article lay the groundwork for future analyses involving different breeds, diets, and environmental conditions. By applying these techniques to other goat populations, researchers could further explore the adaptability and generalizability of the models, thereby expanding their applicability to a wider range of farming contexts.

### 4.2. Comparison with the Previous Study

While the previous study employed regression models, access to the predictions generated by the linear regression models used in that study was not available. This limitation prevented the calculation of key performance metrics, such as MAE or MSE, which are the focus of Section 3.1. Consequently, a direct comparison based on these criteria is not feasible, restricting the ability to perform a detailed analysis of the regression models’ predictive performance under identical conditions.

However, examining the results obtained from factored data, corresponding to those in Section 3.2, the original study reported moderate classification performance. Accuracy and sensitivity were relatively high, but specificity and NPV were notably lower. These metrics serve as a useful reference point to assess the improvements observed with the methods applied in this study, despite the lack of error measures from the original models.

In contrast to the original study’s regression models [13], the decision tree model employed here demonstrated marked improvements in classification performance. Specifically, the decision tree achieved an accuracy of 0.9816, a sensitivity of 1.0, a specificity of 0.9741, and an NPV of 1.0. These results represent a substantial enhancement in both the identification of positive and negative cases compared to the original study, which achieved an accuracy of 0.8745, a sensitivity of 0.9303, a specificity of 0.7143, and an NPV of 0.78125. The high sensitivity and NPV values in the decision tree model underscore its superior performance, particularly in avoiding false negatives.

Similarly, the neural network model showed notable improvements, with an accuracy of 0.9632, a sensitivity of 0.94, a specificity of 0.9735, and an NPV of 0.9735. While its sensitivity is slightly lower than that of the decision tree model, it still surpasses the original regression model across all metrics. The high specificity and NPV values, combined with strong accuracy, further highlight the effectiveness of machine learning techniques such as neural networks in handling complex biological data. These advancements underscore the reliability of these models in more effectively predicting both positive and negative cases, leading to more accurate and consistent outcomes.

### 4.3. Advancements Through Machine Learning (ML) and Deep Learning (DL) in Biological Data

The application of data science, particularly through ML and DL, has enhanced the analysis of biological data, offering significant advancements over traditional statistical techniques. While classical methods such as linear regression or logistic models have long been used to model biological processes, their limitations in handling complex, high-dimensional datasets have become increasingly evident. In contrast, ML and DL algorithms excel at identifying intricate patterns and relationships within large datasets, providing more accurate and nuanced predictions. These techniques enable models to capture non-linear interactions that traditional approaches often overlook, resulting in improved precision and reliability in biological research.

Recent publications have consistently demonstrated the benefits of employing ML and DL in the animal sciences, with numerous examples illustrating their capacity to enhance predictive accuracy and uncover hidden insights [38,39,40]. From genomics and proteomics to disease prediction and animal health monitoring, these advanced methods have allowed for researchers to tackle previously intractable problems [41]. The use of ML and DL has led to breakthroughs in areas such as biomarker discovery, diagnostic tool development, and precision farming, showcasing their transformative potential across diverse biological fields [42,43]. This shift towards data-driven methodologies marks a significant evolution in the way biological data are analyzed and applied, offering new opportunities for innovation and efficiency.

A growing body of research demonstrates the better performance of ML and DL algorithms compared to traditional statistical approaches like multiple linear regression (MLR) in the analysis of complex biological datasets. For instance, Chen et al. [44] compared the prediction of nitrogen (N) excretion in lactating dairy cows found that ML or DL techniques such as artificial neural networks (ANNs), random forest regression (RFR), and support vector regression (SVR) outperformed MLR models in both accuracy and precision. In this study, the ANN model achieved significantly lower RMSE and higher concordance correlation coefficient (CCC) compared to the MLR model, underscoring the capacity of DL algorithms to handle the non-linear relationships and complex interactions present in biological systems. Thus, Chen et al. [44] highlights an important challenge inherent to MLR models: their reliance on assumptions such as linearity, homoscedasticity, and normality of residuals, which may not always hold true in complex datasets. The authors note that MLR models, while useful in certain contexts, can lead to biased results or fail to provide satisfactory predictions when these assumptions are violated. In contrast, ML models, and particularly ANN models, demonstrated the ability to explore deeper relationships between the variables and outputs, improving the predictive power without the need for strict assumptions about the data. These findings align with broader trends in biological research, where the shift towards data-driven methodologies has become essential for dealing with increasingly complex datasets.

Moreover, the study developed new ANN models for the prediction of manure nitrogen excretion. These models were able to predict with greater accuracy than traditional MLR methods. Notably, the ANN model produced a lower RMSE and higher CCC compared to the MLR, reflecting its superior ability to generalize across the dataset. This is a critical advantage when applied to practical situations in dairy farms, where accurate predictions of nitrogen excretion are necessary to mitigate environmental impacts and enhance economic sustainability.

The study by Hansen et al. [45] explores how modern ML, DL, and statistical methods can enhance the forecasting of milk deliveries to dairy plants compared to traditional techniques. Using historical data from Norwegian dairy farms, the authors evaluated several models, including seasonal ARIMA, LASSO, Group LASSO, the Prophet model, Boosting, and neural network autoregression (NNAR). The primary goal was to improve prediction accuracy over different time horizons, ranging from 1 to 24 months. The results demonstrated that ML and DL models, such as Boosting and NNAR, significantly outperformed traditional statistical methods in long-term forecasting, especially by identifying key features that influence future milk deliveries, such as the number of cows, inseminations, and calvings per month.

In particular, the NNAR model produced the best results for 12-month forecasts, while the SARIMAX model was more accurate for 24-month predictions. The study highlights that data-driven models like Boosting provided greater flexibility and accuracy, especially in long-term predictions, due to their ability to handle non-linear relationships and complex features in the data.

Similar to the present study, Hansen et al. [45] demonstrates that integrating ML and DL techniques offers significant improvements over traditional multiple linear regression models. While conventional approaches like ARIMA and the Prophet model remain useful for short-term predictions, more advanced models such as Boosting and neural networks deliver superior predictive capability, particularly when dealing with complex, multivariate datasets.

Hu et al. [43] investigated the application of ML techniques in combination with hyperspectral imagery from unmanned aerial vehicles (UAVs) to estimate the biomass of milk vetch, a winter-growing cover crop known for its ability to enhance soil fertility. The authors compared several regression models—random forest (RF), MLR, support vector machine (SVM), and deep neural network (DNN)—to evaluate their performance in biomass prediction. The research found that RF, which is an ensemble method based on decision trees, achieved the highest coefficient of determination (R^2^ = 0.950) and the lowest relative root mean square error (RRMSE = 14.86%) among all models. Notably, the DNN model also performed well on the test set, slightly surpassing RF in some respects, such as its performance in the second year of data collection.

The study highlights the significant potential of combining UAV-based hyperspectral data with ML techniques to perform large-scale, non-invasive biomass estimations. Hyperspectral imagery allowed for the researchers to compute vegetation indices (VIs) that served as inputs to the ML models. Through feature selection using Pearson correlation and principal component analysis (PCA), the VIs most strongly correlated with biomass were identified, ensuring that the models were built with the most relevant predictors. The results demonstrated that ML models, particularly RF and DNN, provide accurate predictions, significantly outperforming traditional methods like MLR in biomass estimation.

The parallels between the findings from these studies and our research further reinforce the growing consensus that ML and DL techniques offer a transformative approach to biological data analysis. By surpassing the limitations of traditional MLR, these advanced methods not only improve predictive accuracy but also provide deeper insights into the complex relationships between variables, such as those observed in animal health, agricultural productivity, and environmental management. As the integration of ML and DL models becomes increasingly prevalent in biological research, it is evident that these tools are indispensable for advancing both scientific understanding and practical applications in fields that rely on accurate, large-scale data analysis.

### 4.4. Future Implications

As described above, the determination of IgG concentration in goat colostrum is crucial, for ensuring the survival of newborn kids. High circulating IgG concentration is vital for providing adequate immunity to these animals during their early stages of life. However, while accurate IgG measurements are essential, precise laboratory methods are often costly and inaccessible for many farmers, who are forced to rely on less accurate farm-based techniques [8]. These alternative methods, while more affordable, do not offer the same level of precision, leading to potential risks in the health and survival of young animals.

The results of this study demonstrated that both the decision tree and neural network models are able to predict the IgG concentration that closely approximate to those obtained through laboratory testing. This means that by implementing these models through a simple and cost-effective system, farmers could achieve reliability levels exceeding 95%. Such an approach would provide farmers with a practical solution that bridges the gap between accuracy and affordability, without the need for expensive lab process.

Moreover, these models can be continuously refined and improved by integrating more data over time, further enhancing their reliability. They are designed to be user-friendly and can be implemented in practical farm settings through integration into portable devices or user-friendly software. This would enable farmers to measure colostrum quality effectively with minimal technical expertise. The data required to operate these models can be readily collected using accessible tools, ensuring that even small-scale farmers can benefit from their use. This scalability and adaptability make these models a highly valuable tool for ensuring livestock health and well-being, contributing to more sustainable and efficient farm management practices in the future.

## 5. Conclusions

In this study, AI techniques were successfully integrated to evaluate IgG concentration in goat colostrum using a low-cost color method. By employing both ML and DL methodologies, specifically regression based on decision trees and neural networks, models were developed to provide reliable support for goat farms without incurring the high costs associated with traditional laboratory techniques. The results indicate that the decision tree regression model outperformed the neural network model across multiple metrics.

These findings suggest that the decision tree regression model provides a highly accurate and reliable method for assessing IgG concentration, offering a cost-effective solution for goat farms.

## Figures and Tables

**Table 1:** Contingency tables for decision tree regression model.

	**HIGH**	**LOW**	
**HIGH**	**47**	**3**	**50**
**LOW**	**0**	**113**	**113**
	**47**	**116**	

**Table 2:** Contingency tables for neural network regression model.

	**HIGH**	**LOW**	
**HIGH**	**47**	**3**	**50**
**LOW**	**3**	**110**	**113**
	**50**	**113**	

## Data Availability

The data presented in this study are available in Appendix A of this article.

## Appendix A

**Table A1:** Original and predicted values from decision tree and neural network regression models.

L	Cr	Hue	IgG	IgG_p_DT	IgG_p_NN
83.86	28.40	103.56	28.71	28.71	30.98
94.09	7.68	113.23	3.97	3.97	3.59
90.34	23.28	107.14	24.30	24.30	24.78
94.62	6.48	106.67	1.78	1.78	1.45
77.88	21.54	99.76	38.19	38.19	39.41
89.33	18.90	105.50	32.96	32.96	33.40
90.17	11.12	111.03	19.69	19.69	19,00
90.98	10.59	114.24	1.92	1.92	0.90
88.66	8.48	107.07	15.48	15.48	15.49
92.59	8.70	101.80	12.80	12.80	12.45
91.00	8.00	108.00	5.00	5.00	4.40
89.89	21.57	106.10	24.62	24.62	25.03
87.97	18.06	108.19	16.87	16.87	16.82
87.01	20.36	105.11	37.40	37.40	38.65
88.73	24.22	106.38	33.30	33.30	34.01
91.10	18.47	108.83	20.71	20.71	20.39
88.45	15.92	105.55	16.80	16.80	16.81
93.12	11.32	108.48	8.32	8.32	7.29
93.50	10.03	105.26	4.60	4.60	3.93
84.68	16.76	105.82	33.94	33.94	33.87
90.36	8.32	100.39	11.42	11.42	11.54
91.08	11.01	107.17	5.08	5.08	4.59
90.31	6.56	109.40	4.75	4.75	4.26
89.71	9.95	110.60	6.65	7.52	20.94
92.00	9.00	108.00	3.00	3.00	2.89
94.52	9.95	111.90	2.55	2.55	1.49
91.00	18.00	108.00	18.00	18.00	18.03
90.00	15.00	110.00	22.00	22.00	22.23
88.00	15.00	106.00	22.00	22.00	21.65
93.03	7.30	110.77	6.00	6.00	4.64
92.00	11.00	113.00	9.00	9.00	8.15
92.89	8.90	110.59	7.52	7.52	7.20
87.33	20.62	101.95	24.44	24.44	25.02
89.94	11.12	108.68	17.38	17.38	16.08
91.65	12.39	108.98	14.47	14.47	14,00
90.98	10.59	114.24	1.92	1.92	0.90
88.89	13.97	101.23	6.16	6.16	5.58
91.46	9.25	111.24	0.50	0.50	0.52
91.68	11.21	111.39	4.91	4.91	4.02
91.28	9.24	110.40	18.79	18.79	17.41
92.00	7.00	111.00	5.00	5.00	3.90
92.00	10.00	111.00	15.00	13.33	12.65
87.97	18.06	108.19	16.87	16.87	16.82
91.66	8.66	115.50	11.37	11.37	10.28
90.00	15.00	110.00	22.00	22.00	22.23
90.00	20.00	107.00	22.00	22.00	22.47
83.76	21.91	102.23	33.64	33.64	34.89
75.00	29.00	98.00	32.00	32.00	34.56
90.45	9.62	111.92	10.83	10.83	10.51
94.52	9.95	111.90	2.55	2.55	1.49
90.87	15.93	105.62	11.51	11.51	12.84
92.60	8.67	114.02	11.42	11.42	8.59
91.75	8.54	114.04	4.26	4.26	3.75
84.62	18.87	105.15	11.21	11.21	11.86
94.23	7.87	113.60	2.34	2.34	2.19
92.45	9.05	107.81	13.05	13.05	11.07
85.08	13.08	101.34	6.67	6.67	5.92
89.71	9.95	110.60	6.65	7.52	20.94
93.92	7.53	109.23	3.05	3.05	2.75
93.36	6.30	113.68	1.03	1.03	2.37
91.16	8.84	109.30	10.72	10.72	9.13
89.71	9.95	110.60	6.65	7.52	20.94
88.34	11.48	103.81	9.89	9.89	8.96
88.66	8.48	107.07	15.48	15.48	15.49
88.07	18.24	105.00	24.36	24.36	25.37
88.58	15.82	106.68	14.85	14.85	14.86
92.56	7.74	116.63	1.30	1.30	0.98
92.61	8.69	109.64	4.08	4.08	3.98
93.04	7.00	113.49	7.26	7.26	7.29
90.61	14.80	109.59	13.23	13.23	14.38
89.36	11.86	108.18	10.84	10.84	9.11
86.66	18.03	102.13	23.45	23.45	23.87
92.56	9.35	110.22	4.46	4.46	5.48
88.69	19.18	105.33	36.35	36.35	37.29
92.97	5.41	107.87	2.60	2.60	2.43
88.97	7.55	114.43	2.56	2.56	2.46
88.36	20.44	105.58	22.79	22.79	23.76
85.01	9.32	101.77	0.82	0.82	-0.45
87.36	14.40	103.99	8.03	8.03	7.59
91.12	7.63	114.89	8.46	8.46	8.47
88.00	20.00	106.00	22.00	22.00	23.04
88.94	9.46	97.16	14.31	14.31	13.85
84.68	16.76	105.82	33.94	33.94	33.87
90.83	13.84	107.23	5.44	5.44	5.15
92.00	7.00	115.00	6.00	6.00	3.92
86.07	20.07	109.20	23.44	23.44	23.58
87.26	21.4	105.06	28.10	28.10	31.99
93.00	8.11	117.07	6.14	6.14	4.22
86.24	11.53	104.77	4.72	4.72	3.38
92.75	6.49	110.28	4.48	4.48	3.80
91.62	23.87	105.83	32.37	32.37	32.73
84.62	18.87	105.15	11.21	11.21	11.86
88.55	13.03	108.02	5.51	5.51	4.65
93.31	10.57	107.51	7.04	7.04	6.75
89.83	6.93	123.23	6.92	6.92	5.15
91.71	15.88	111.07	22.54	8.77	21.10
93.92	7.53	109.23	3.05	3.05	2.75
93.50	9.58	107.68	11.40	11.40	10.91
88.55	13.03	108.02	5.51	5.51	4.65
88.68	23.52	105.11	22.35	22.35	23.11
92.00	9.00	108.00	3.00	3.00	2.89
92.56	9.35	110.22	4.46	4.46	5.48
96.75	21.11	104.17	23.06	23.06	18.93
92.72	13.89	109.30	10.60	10.60	10.76
83.76	21.91	102.23	33.64	33.64	34.89
89.50	18.99	103.49	23.98	23.98	23.67
94.68	11.24	108.47	7.27	7.27	5.22
93.24	8.08	115.11	13.80	13.80	8.19
87.22	7.59	111.66	5.87	5.87	5.60
91.32	11.58	109.58	20.27	20.27	19.79
91.08	9.32	106.45	5.09	5.09	5.44
93.82	17.25	106.99	25.06	25.06	23.91
89.80	18.5	107.26	22.38	22.38	22.54
98.53	9.27	105.27	3.51	3.51	1.40
90.99	17.46	107.60	25.35	25.35	25.33
88.37	18.39	103.30	17.51	17.51	18.40
88.53	11.77	102.47	6.51	6.51	6.10
93.87	9.90	108.07	1.03	1.03	0.82
91.32	13.65	106.07	18.38	18.38	17.65
83.28	15.55	103.72	8.43	8.43	8.94
93.58	11.45	109.34	14.51	14.51	12.83
91.28	11.33	107.89	22.45	22.45	21.75
89.50	18.99	103.49	23.98	23.98	23.67
82.19	23.73	100.27	34.00	34.00	36.05
89.33	18.90	105.50	32.96	32.96	33.40
92.00	10.00	111.00	10.00	13.33	12.65
92.00	10.88	110.22	9.53	9.53	9.08
92.00	11.00	113.00	9.00	9.00	8.15
91.71	15.88	111.07	22.54	8.77	21.10
91.17	7.89	112.83	2.38	2.38	2.53
92.00	5.00	110.00	3.00	3.00	2.30
91.39	7.54	123.58	5.41	5.41	2.51
88.00	17.00	101.00	28.00	28.00	29.07
90.09	23.46	107.85	25.97	25.97	26.55
88.89	13.97	101.23	6.16	6.16	5.584
92.03	15.42	109.95	8.77	8.77	8.59
91.80	8.75	106.88	3.83	3.83	3.77
89.25	18.46	107.11	28.49	28.49	28.13
91.02	18.30	106.90	13.07	13.07	13.49
92.80	8.24	109.86	4.07	4.07	3.65
91.12	7.63	114.89	8.46	8.46	8.47
92.31	7.85	112.78	8.98	8.98	9.34
79.99	22.05	98.56	22.85	22.85	23.76
90.24	10.20	110.42	19.73	19.73	19.05
93.46	10.48	109.33	4.68	4.68	3.93
88.13	27.58	106.92	31.66	31.66	32.91
85.24	10.87	105.15	0.90	0.90	0.33
90.90	12.23	109.00	15.46	15.46	15.13
94.69	8.77	107.73	7.43	7.43	6.35
93.38	7.99	115.57	4.67	4.67	5.12
93.82	17.25	106.99	25.06	25.06	23.91
91.74	12.05	109.34	7.22	7.22	7.64
90.31	6.56	109.40	4.75	4.75	4.26
91.52	6.39	120.38	0.70	0.70	-0.11
90.00	14.00	105.00	20.00	20.00	19.71
91.70	12.93	110.13	17.20	17.20	17.07
75.00	29.00	98.00	32.00	32.00	34.56
62.54	28.82	98.94	31.39	31.39	32.34
92.95	10.73	114.27	11.27	11.27	9.60
90.00	10.00	100.00	15.00	15.00	14.56
91.71	15.88	111.07	22.54	8.77	21.10
83.86	28.40	103.56	28.71	28.71	30.98
92.00	10.00	111.00	10.00	13.33	12.65
